# Safety and Efficacy of Subcutaneous Rituximab in Previously Untreated Patients with CD20+ Diffuse Large B-Cell Lymphoma or Follicular Lymphoma: Results from an Italian Phase IIIb Study

**DOI:** 10.1155/2022/5581772

**Published:** 2022-01-27

**Authors:** Mario Petrini, Gianluca Gaidano, Andrea Mengarelli, Ugo Consoli, Armando Santoro, Anna Maria Liberati, Marco Ladetto, Vincenzo Fraticelli, Attilio Guarini, Donato Mannina, Paola Ferrando, Paolo Corradini, Pellegrino Musto, Caterina Stelitano, Dario Marino, Andrea Camera, Marco Murineddu, Roberta Battistini, Giuseppe Caparrotti, Mauro Turrini, Luca Arcaini, Simone Santini, Manuela Cerqueti, Andres J. M. Ferreri, Nicola Cantore, Alessandro Inzoli, Giovanni Cardinale, Benedetto Ronci, Giorgio La Nasa, Stefano Massimi, Gianfranco Gaglione, Valentina Barbiero, Maurizio Martelli

**Affiliations:** ^1^Section of Hematology, Department of Clinical and Experimental Medicine, University of Pisa, Pisa, Italy; ^2^Division of Hematology, Department of Translational Medicine, University of Eastern Piedmont, Novara, Italy; ^3^Hematology Unit, Experimental and Clinical Oncology Department, IRCCS Regina Elena National Cancer Institute, Rome, Italy; ^4^Division of Hematology, ARNAS Garibaldi, Catania, Italy; ^5^Humanitas Clinical and Research Center-IRCCS, Humanitas Cancer Center, Rozzano, Milan, Italy; ^6^Humanitas University Pieve Emanuele, Milan, Italy; ^7^Azienda Ospedaliera “Santa Maria”, Terni, Italy; ^8^Hematology Division, Azienda Ospedaliera Santi Antonio e Biagio e Cesare Arrigo, Alessandria, Italy; ^9^Department of Translational Medicine, University of Eastern Pedmont, Alessandria, Italy; ^10^Department of Hematology, Gemelli Molise, Campobasso, Italy; ^11^Hematology and Cell Therapy Unit, IRCCS-Istituto Tumori ‘Giovanni Paolo II', Bari, Italy; ^12^Hematology Unit, Azienda Ospedaliera, Papardo, Messina, Italy; ^13^Medical Oncology, ASST Lecco, Lecco, Italy; ^14^Fondazione IRCCS Instituto Nazionale dei Tumori, University of Milan, Milano, Italy; ^15^Department of Emergency and Organ Transplantation “Aldo Moro”, University School of Medicine and Unit of Hematology and Stem Cell Transplantation, AOU Consorziale Policlinico, Bari, Italy; ^16^Division of Haematology, Azienda Ospedaliera Bianchi-Melacrino-Morelli, Reggio Calabria, Italy; ^17^Department of Clinical and Experimental Oncology, Oncology Unit 1, Veneto Institute of Oncology IOV-IRCCS, Padua, Italy; ^18^Hematology Unit, Sant'Anna e San Sebastiano Hospital, Caserta, Italy; ^19^Hematology & Bone Marrow Transplant Unit, San Francesco Hospital, Nuoro, Italy; ^20^Hematology Unit, AO San Camillo Forlanini, Rome, Italy; ^21^Department of Haematology, ASL Caserta (CE), Hospital Moscati, Aversa (CE), Italy; ^22^Division of Hematology, Valduce Hospital, Como, Italy; ^23^Division of Hematology, Fondazione IRCCS Policlinico San Matteo and Department of Molecular Medicine, University of Pavia, Italy; ^24^ASL Toscana Centro, Department of Oncology, Oncoematology Unit, Santo Stefano Hospital, Prato, Italy; ^25^General Medicine, Hospital of Macerata, Macerata, Italy; ^26^Department of Onco-Haematology, Division of Experimental Oncology, IRCCS San Raffaele Hospital, Milan, Italy; ^27^Hematology and Hematopoietic Transplantation Unit, San G. Moscati Hospital, Avellino, Italy; ^28^Hematology Unit, Ospedale Maggiore ASST, Crema, Italy; ^29^Onco-hematology Unit, ARNAS Civico Di Cristina Benfratelli, Palermo, Italy; ^30^Hematology Unit, SS Filippo and Nicola Hospital, ASL1 Avezzano (AQ), Avezzano, Italy; ^31^Department of Medical Science and Public Health, Hematology and Transplant Center, “A. Businco” Hospital, University of Cagliari, Cagliari, Italy; ^32^Roche S. p. A., Monza, Italy; ^33^Department of Translational and Precision Medicine, Sapienza University, Rome, Italy

## Abstract

Subcutaneous (SC) rituximab may be beneficial in terms of convenience and tolerability, with potentially fewer and less severe administration-related reactions (ARRs) compared to the intravenous (IV) form. This report presents the results of a phase IIIb study conducted in Italy. The study included adult patients with CD20+ DLBCL or FL having received at least one full dose of IV RTX 375 mg/m^2^ during induction or maintenance. Patients on induction received ≥4 cycles of RTX SC 1400 mg plus standard chemotherapy and FL patients on maintenance received ≥6 cycles of RTX SC. Overall, 159 patients (73 DLBCL, 86 FL) were enrolled: 103 (54 DLBCL, 49 FL) completed induction and 42 patients with FL completed 12 maintenance cycles. ARRs were reported in 10 patients (6.3%), 3 (4.2%) with DLBCL and 7 (8.1%) with FL, all of mild severity, and resolved without dose delay/discontinuation. Treatment-emergent adverse events (TEAEs) and serious adverse events occurred in 41 (25.9%) and 14 patients (8.9%), respectively. Two patients with DLBCL had fatal events: *Klebsiella* infection (related to rituximab) and septic shock (related to chemotherapy). Neutropenia (14 patients, 8.9%) was the most common treatment-related TEAE. Two patients with DLBCL (2.8%) and 6 with FL (7.0%) discontinued rituximab due to TEAEs. 65.2% and 69.7% of patients with DLBCL and 67.9% and 73.6% of patients with FL had complete response (CR) and CR unconfirmed, respectively. The median time to events (EFS, PFS, and OS) was not estimable due to the low rate of events. At a median follow-up of 29.5 and 47.8 months in patients with DLBCL and FL, respectively, EFS, PFS, and OS were 70.8%, 70.8%, and 80.6% in patients with DLBCL and 77.9%, 77.9%, and 95.3% in patients with FL, respectively. The switch from IV to SC rituximab in patients with DLBCL and FL was associated with low risk of ARRs and satisfactory response in both groups. This trial was registered with NCT01987505.

## 1. Introduction

Non-Hodgkin's lymphomas (NHL) are a heterogeneous group of lymphoproliferative malignancies and are one of the leading causes of cancer death in both the United States and Europe [1]. Diffuse large B-cell lymphoma (DLBCL) is the most common histologic subtype of NHL and represents approximately 25% of new NHL cases each year [2]. The incidence of DLBCL increases with advancing age and represents more than half of NHL cases among patients above 65 years of age [3] who have a worse prognosis compared to younger patients [4]. Follicular lymphoma (FL) is the second most frequent subtype of lymphoid malignancies in Western Europe, and its annual incidence has been increasing in recent years [5].

Rituximab is a chimeric murine/human monoclonal antibody that specifically binds to CD20, a hydrophobic transmembrane protein expressed on the surface of B lymphocytes [6]. Rituximab has been shown to induce both complement-mediated and antibody-dependent cell mediated lyses of CD20+ cells [7]. It has also been reported to have direct antitumour activity, as indicated by the induction of apoptosis of human B-cell lines [8]. In addition, it sensitizes drug-resistant human B-cell lymphoma cell lines to the cytotoxic effects of some chemotherapeutic agents [9].

Rituximab was first developed as a solution for intravenous (IV) administration and was approved in 1997 for the treatment of NHL at a dose of 375 mg/m^2^. The use of IV rituximab has become standard in the management of patients suffering from various B-cell malignancies, including FL and DLBCL. In these diseases, several randomised clinical trials have demonstrated that rituximab, administered as monotherapy or in combination with chemotherapy, not only prolongs the time to disease progression but also extends overall survival [10].

From a safety perspective, a cluster of signs and symptoms reported during or within 24 hours of rituximab IV infusion, which may be related to the release of cytokines and/or other chemical mediators, has been well characterised in pivotal trials of IV rituximab and from postmarketing experience. Infusion-related reactions (IRRs) were identified as the most common adverse drug reactions in patients treated with IV rituximab as monotherapy or combination therapy [10]. Such IRRs may require prolonging the infusion time. Furthermore, the required procedure to establish IV access is considered invasive and may cause discomfort, particularly in patients with malignant diseases undergoing repeated and frequent treatments. A subcutaneous (SC) formulation of rituximab has been developed to address these limitations (i.e., infusion/injection-related reactions [IIRRs], long administration time, hospital facility requirements, and difficulty treating patients with poor venous access). Moreover, SC administration of rituximab takes significantly less time (5-6 minutes) compared to IV infusion and this is expected to improve treatment convenience, patient satisfaction, and compliance, as well as reduced work time for the staff. Comparative trials of IV and SC rituximab have shown that administration by the SC route is associated with noninferior pharmacokinetics [11] and comparable efficacy to the IV route, with no new safety concerns [12,13]. The evidence from clinical trials has led to the approval of SC rituximab for the treatment of patients with DLBCL, FL, and chronic lymphocytic leukaemia.

Here, we present results from an Italian, open-label, single-arm, phase IIIb trial conducted in three regions (Italy, Spain, and North Africa) aimed at evaluating the safety, efficacy, and patient satisfaction of switching rituximab administration from the IV to the SC route in patients with DLBCL and FL.

## 2. Materials and Methods

### 2.1. Patients

The study population included patients of either sex aged 18–80 years, with histologically confirmed CD20+ DLBCL or grade 1-3a follicular NHL (FL), currently being treated with IV rituximab as first-line induction/maintenance and having received at least one full dose of IV rituximab (375 mg/m^2^) administered without interruption and can receive ≥4 additional induction cycles (DLBCL/FL) or ≥6 maintenance cycles (FL). To be eligible for the study, patients were also required to have the following: an Eastern Cooperative Oncology Group (ECOG) performance status ≤3; an International Prognostic Index (IPI) score of 1–4 or IPI score of 0 with bulky disease, defined as one lesion ≥7.5 cm, or Follicular Lymphoma International Prognostic Index (FLIPI) score (low, intermediate, or high risk) assessed before the first rituximab IV administration in induction; and at least one bidimensionally measurable lesion defined as ≥1.5 cm in its largest dimension on computed tomography (CT) scan assessed up to 45 days before the first rituximab IV administration in induction.

Patients with any of the following conditions were excluded from the study: transformed lymphoma or FL grade 3b; primary central nervous system lymphoma, histologic evidence of transformation to Burkitt lymphoma, primary effusion lymphoma, primary mediastinal DLBCL, DLBCL of the testis, or primary cutaneous DLBCL; history of other malignancies that could affect compliance with the protocol or interpretation of results; history of HIV positivity; ongoing corticosteroid use >30 mg/day of prednisone or equivalent (a prephase of high dose prednisolone was acceptable for patients with aggressive NHL); inadequate renal, hepatic, or haematologic function; active hepatitis B or C; contraindication to any of the individual components of CHOP (cyclophosphamide, vincristine, doxorubicin, and prednisone) in DLBCL patients; history of severe allergic or anaphylactic reactions to humanized or murine monoclonal antibodies or known sensitivity or allergy to murine products; active and/or severe infections requiring treatment with IV antibiotics or hospitalization within 4 weeks prior to treatment; life expectancy <6 months; pregnant or lactating females or women of childbearing potential not using an effective measure of contraception.

### 2.2. Study Procedures

Patients receiving induction/maintenance IV rituximab were switched to SC rituximab 1400 mg (irrespective of patient body surface area), that is, an injection volume of 11.7 ml, on an outpatient setting. No dose modification for SC rituximab was allowed. Patients received premedication with paracetamol and diphenhydramine, or alternative antihistamines, 30–60 minutes prior to rituximab administration. Rituximab was administered prior to standard chemotherapy regimen, which was CHOP-21 (cyclophosphamide, vincristine, doxorubicin, and prednisone) or CHOP-14 in DLBCL and CHOP-21 or CVP (cyclophosphamide, vincristine, and prednisone) or bendamustine in FL (induction only).

Patients on induction prior to entry into the study received ≥4 cycles of SC rituximab (i.e., 4 additional months of treatment) plus standard chemotherapy and those who were continuing on maintenance after final staging during the study could continue to receive SC rituximab up to 12 cycles. Patients on maintenance prior to entry into the study received ≥6 cycles of SC rituximab (i.e., 12 months of treatment) and those who were continuing on maintenance following at least 4 cycles of SC rituximab during induction could receive ≥6 further cycles of SC rituximab. Patients who completed induction with IV rituximab could be enrolled in the maintenance therapy of the study starting from cycle 1 with SC rituximab. FL patients who achieved at least a partial response at weeks 4–6 after induction were eligible for maintenance with single-agent SC rituximab. All patients who completed the study treatment then entered the posttreatment follow-up phase until the end of the study, in which study visits were placed every 3 and 6 months (±2 weeks) during the first and second year, respectively.

### 2.3. Outcome Measures

The primary study endpoint was the incidence of administration-related reactions (ARRs), defined as all related AEs occurring within 24 hours of SC rituximab administration, including IIRRs, injection-site reactions, administration site conditions, and all symptoms thereof. Other safety endpoints included the incidence of treatment-emergent adverse events (TEAEs), grade ≥3 TEAEs and ARRs, serious adverse events (SAEs), safety laboratory tests (haematology, biochemistry, and coagulation parameters), and ECOG performance status. Efficacy endpoints were the following: tumor response measured 4–8 weeks after the end of induction; event-free survival (EFS), defined as the time from the first dose to the first occurrence of progression or relapse, or initiation of a non-protocol-specified antilymphoma therapy or death, whichever occurred first; progression-free survival (PFS); and overall survival (OS). Moreover, patients' satisfaction with treatment was based on the Rituximab Administration Satisfaction Questionnaire (RASQ).

### 2.4. Statistics

The sample size calculation was based on data from a previous study (Salar et al., 2014), which showed that the expected proportion of ARRs after SC rituximab was approximately 30%. A sample size of 160 patients would have assured that the precision of the estimate would be ±7.2%, so the confidence interval (CI) would have ranged from 22.8% to 37.2%.

TEAE terms were assigned to a preferred term (PT) and were classified by primary system organ class (SOC) according to MedDRA version 19.1. The proportion of patients experiencing at least one ARR, TEAE, and SAE was estimated with its 95% Clopper-Pearson CI. Results of time to event variables were evaluated by means of Kaplan-Meier estimates of the median time to event and the corresponding 2-sided 95% CI. Results of RASQ were summarised by count and percentage and presented overall and by subgroup of diagnosis (DLBCL and FL). RASQ domain scores were expressed as [mean of completed item responses-1] × 25, where the maximum possible item response (best) value was 5 and the minimum possible response value (worst) was 1.

### 2.5. Ethics

The study protocol was approved by the reference Ethic Committee of each investigational study site prior to study start. Patients gave their written informed consent to participate in the study prior to the start of any study-related procedure.

## 3. Results

### 3.1. Patient Disposition and Baseline Characteristics

A total of 159 patients were enrolled in 37 sites in Italy and there were 15 screening failures. The disposition of patients is summarised in Table 1. Of the 159 enrolled patients, 73 had DLBCL and 86 had FL. One enrolled patient with DLBCL did not start treatment and was excluded from the analysis. A total of 67 patients (42.1% of enrolled) with DLBCL (91.8% in this subgroup) completed the final staging, and 77 patients (48.4% of enrolled) with FL (89.5% in this subgroup) continued to the maintenance phase. Overall, 38 patients (23.9%), 13 (17.8%) with DLBCL and 25 (29.1%) with FL, prematurely discontinued treatment. Disease progression, with 7 (53.8%) patients with DLBCL and 7 (28.8%) with FL, and AEs, with 2 (15.4%) patients with DLBCL and 10 (40.0%) with FL, were the most common reasons for treatment discontinuation.

Table 2 shows the demographic and baseline characteristics of patients. Patients with DLBCL were predominantly males, while the proportions of male and female patients were similar. The majority of patients were aged ≥18 and ≤64 years (107 patients, 67.7%). Most of the patients (131 patients, 82.9%) were of non-Hispanic ethnicity. Most of the patients with DLBCL had an ECOG performance status of grade 0 (36 patients, 50.0%) or grade 1 (30 patients, 41.7%), while grade 0 ECOG performance status was predominant in patients with FL (71 patients, 82.6%).

### 3.2. Study Treatment and Concomitant Therapy

All patients received a full dose of SC rituximab 1400 mg at any cycle. The mean (±SD) number of cycles of rituximab SC was 5.9 ± 1.47 (median 6.0, range 1–7) in patients with DLBCL and 12.5 ± 5.30 (median 12.0, range 2–19) in patients with FL. Of patients with FL, 49 patients (57.0%) completed 5, 6, or 7 cycles of induction, 24 (27.9%) completed 7 cycles of induction, and 42 (48.8%) completed 12 cycles of maintenance. Of patients with DLBCL, 54 patients (75.0%) completed 6 or 7 cycles of treatment, and 33 patients (45.8%) completed 7 cycles of treatment.

18 patients with FL (20.9%) received bendamustine, for a mean (±SD) number of 5.1 ± 0.76 (median 5.0, range 3–6) cycles. CHOP was given in 72 (100%) patients with DLBCL and in 33 (38.4%) with FL, for a mean (±SD) number of 4.6 ± 1.56 (median 5.0, range 1–7) and 4.0 ± 1.13 (median 4.0, range 2–6) cycles, respectively, in the two subgroups. 2 patients with FL (2.3%) received CVP. 6 patients with DLBCL (8.3%) and 6 (7.0%) with FL received some cycles of chemoimmunotherapy. 11 patients with DLBCL (15.3%) and 2 (2.3%) with FL received radiotherapy. Granulocyte colony-stimulating factor was given in 18 patients (25.0%) with DLBCL and in 5 (5.8%) with FL.

### 3.3. Safety

Table 3 shows the overall summary of TEAEs. 10 patients (6.3%) overall, 3 (4.2%) with DLBCL and 7 (8.1%) with FL, had ARRs, which mainly consisted of local adverse effects at the site of injection. Erythema at the site of injection and general erythema (both on 3 patients, all with FL) were the most common ARRs. All ARRs were of mild intensity (grade I NCI CTCAE v4.0) and spontaneously resolved without any action taken with rituximab dose, delay, or frequency of administration.

TEAEs were reported in 61 patients with DLBCL (84.7%) and in 73 (84.9%) patients with FL and were grade ≥3 in 37 (51.4%) and 37 (43.0%) patients, respectively, in the two subgroups. Blood and lymphatic system disorders, with 36 patients (50.0%) with DLBCL and 30 (34.9%) with FL, and general disorders and administration site conditions, with 29 (40.3%) and 35 (40.7%) patients, respectively, in the two subgroups, were the most commonly involved SOCs for TEAEs. Neutropenia was the most common TEAE, reported in 30 (41.7%) patients with DLBCL and in 26 (30.2%) with FL.

Treatment-related TEAEs were reported in 17 (23.6%) patients with DLBCL (28 events) and in 24 (27.9%) with FL (51 events). As shown in Table 4, neutropenia was again the most commonly reported treatment-related TEAE, with 5 (6.9%) patients with DLBCL and 9 (10.5%) with FL. Two patients, both in the DLBCL subgroup (2.7%), had fatal TEAEs, which consisted of *Klebsiella* infection (related to rituximab) and septic shock (related to chemotherapy). Another patient with DLBCL died due to disease progression.

Treatment-emergent SAEs were reported in 26 (36.1%) patients with DLBCL and in 23 (26.7%) with FL. Again, neutropenia was the most commonly reported treatment-emergent SAE, with 15 patients with DLBCL (20.8%) and 13 (15.1%) with FL.

IIRRs were reported in 1 patient (1.4%) with DLBCL and in 7 (8.1%) with FL. Erythema at the site of injection and general erythema (both with 3 patients, all FL) were the most common infusion/injection-related reactions.

Two patients (2.8%) with DLBCL and 6 (7.0%) with FL discontinued rituximab due to TEAEs. The TEAEs related to treatment with rituximab leading to rituximab dose discontinuation consisted of *Pneumocystis jirovecii* pneumonia in 1 patient with FL, folliculitis and vulvovaginitis in 1 patient with FL, neutropenia in 1 patient with FL, pneumonia in 1 patient with DLBCL, and hypokalaemia in 1 patient with DLBCL. 1 patient (1.4%) with DLBCL and 3 (3.5%) with FL discontinued chemotherapy due to TEAEs.

In DLBCL, the proportion of patients with grade 0 ECOG performance status was higher at the final staging (47 out of 68 evaluated patients, 69.1%) than at baseline (36 patients, 50.0%). In patients with FL, the proportion of patients with grade 0 ECOG performance status at the final staging (42 out of 53 evaluated patients, 79.2%) was similar to that at baseline (71 patients, 82.6%).

There were no important changes from baseline in laboratory parameters (haematology, coagulation, and clinical chemistry), except for a decrease in mean platelet count from months 1–6 up to the end of the study, which was more pronounced in patients with DLBCL than in those with FL, and a decrease from baseline in mean lymphocytes count from months 1–6 up to the end of study, which was of similar extent in the two subgroups.

### 3.4. Efficacy

The median follow-up was 34.5 months in the overall population (range 2.6–58.3 months), 29.5 months (range 2.6–36.4 months) in patients with DLBCL, and 47.8 months (range 5.5–58.3 months) in those with FL. Results of tumour response were available in 119 patients overall (66 DLBCL and 53 FL) and showed similar rates of response in patients with DLBCL and FL. Complete response (CR) was observed in 43 patients (65.2%; 95% CI 52.4–76.5%) with DLBCL and in 36 (67.9%; 95% CI 53.7–80.1%) with FL, while CR/complete response unconfirmed (CRu) was observed in 46 patients (69.7%; 95% CI 57.1–80.4) with DLBCL and in 39 (73.6%; 95% CI 59.7–84.7) with FL.

**Figure 1:**
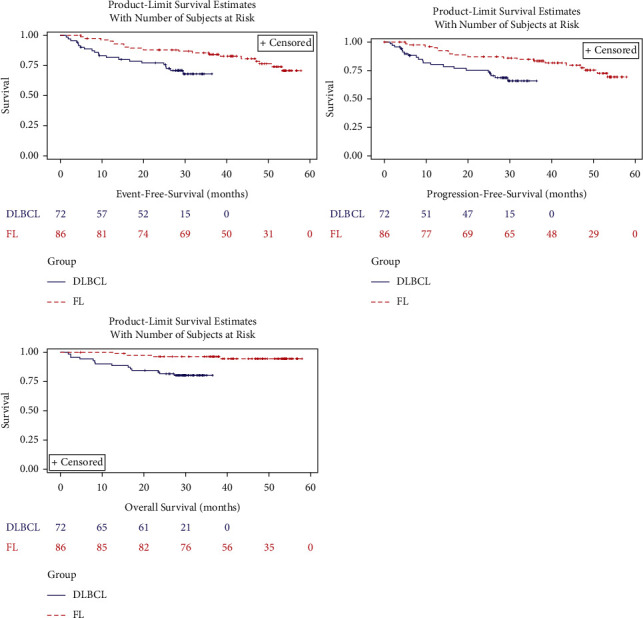
Kaplan–Meier estimate of time to event endpoints (EFS, PFS, and OS). DLBCL: diffuse large B-cell lymphoma; FL: follicular lymphoma; EFS: event-free survival; PFS: progression-free survival; OS: overall survival.

Figure 1 shows the Kaplan-Meier estimate of time to event endpoints (EFS, PFS, and OS). For all variables, the median value was not estimable due to the low number of patients with events. 51 (70.8%) patients with DLBCL and 67 (77.9%) with FL were event-free and progression-free, whereas 21 (29.2%) patients with DLBCL and 19 (22.1%) with FL had events and progression. 58 (80.6%) patients with DLBCL and 82 (95.3%) with FL were alive, whereas 14 (19.4%) patients with DLBCL and 4 (4.7%) with FL died.

### 3.5. Satisfaction with Treatment

**Figure 2:**
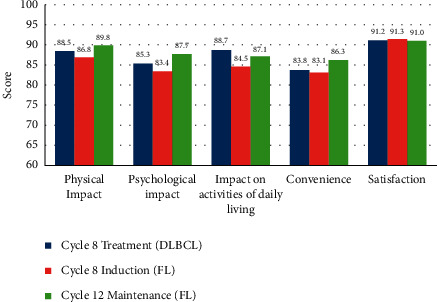
Rituximab Administration Satisfaction Questionnaire: results at cycle 8 treatment (DLBCL patients), cycle 8 induction, and cycle 12 maintenance (FL patients). Labels are mean scores. DLBCL: diffuse large B-cell lymphoma; FL: follicular lymphoma.

The results of the RASQ showed that patients expressed a high level of satisfaction at any time post-baseline in questions regarding satisfaction with the injection, tolerability at the site of injection, expectations for injection, and interference of injections with daily activities (data not shown). For all domains, high mean scores were observed at cycle 8 treatment (patients with DLBCL), cycle 8 induction, and cycle 12 maintenance (patients with FL) (Figure 2).

## 4. Discussion

The results of this Italian phase IIIb clinical study have shown that switching from IV to SC rituximab in patients with DLBCL (73 patients) and FL (86 patients) was associated with a low risk of ARRs and high response rates. The incidence of ARRs was very low, with 6.3% of patients overall, 4.2% of patients with DLBCL, and 8.1% patients with FL that reported ARRs, which mainly consisted of local adverse effects at the site of injection. This rate was lower than that reported in previous clinical trials with SC rituximab in patients with DLBCL [12] and FL [13,14], as well as in the postmarketing setting [15]. Furthermore, all ARRs were of mild intensity and spontaneously resolved without any requirement of changes in rituximab dose or frequency of administration.

Taking into consideration the fact that potential ARRs most frequently occur at the first administration of rituximab [16], the design of this study, which included patients who had previously received at least one dose of IV rituximab, may account for the lower incidence of ARRs compared to head-to-head comparative studies of SC versus IV rituximab. Nonetheless, the recently published results of a “sister” study conducted in Spain (as part of the “MabRella” project), which comprised 29 patients with DLBCL and 111 with FL [17], have shown that ARRs occurred in 48.6% of patients and were of grade ≥3 in 2.1%. Although the design of this study was mainly descriptive and was not aimed at comparing the two DLBCL and FL subgroups, erythema (i.e., the most common ARR in both the Italian and Spanish studies) was reported in more patients with FL than in those with DLBCL in both studies. Therefore, the unbalanced distribution of patients with DLBCL and FL in the Spanish study, compared to the more homogeneous distribution of patients in the two subgroups in the Italian study, may, at least in part, account for the difference in the incidence of ARRs in the two countries. Although the risk of ARRs is high at the first administration of rituximab, the fact that the longer exposure in patients with FL, which was approximately double than that of patients with DLBCL, might have further contributed to the difference in the rate of ARRs in the two subgroups cannot be excluded.

The frequency of TEAEs observed in this study was consistent with the known safety profile of rituximab: neutropenia was the most common TEAE, with 30 (41.7%) patients with DLBCL and 26 (30.2%) with FL, and was considered as related to rituximab in 8.9% of patients overall. In the interpretation of the data, the fact that all patients with DLBCL received CHOP (which was given in less than 40% of patients with FL) and hence chemotherapy may have contributed to haematological toxicity should be taken into consideration. Conversely, the steroidal component of CHOP might have contributed to the lower frequency of cutaneous events (i.e., erythema and injection-site erythema) in patients with DLBCL compared to those with FL.

As further confirmation that adverse reactions were generally well tolerated, only 8 patients overall (5.1%), 2 with DLBCL and 6 with FL, discontinued treatment with rituximab due to TEAEs. There were two deaths due to TEAEs: the case of *Klebsiella* infection was considered as related to treatment with rituximab.

Switching from IV to SC rituximab was associated with high and similar response rates in both subgroups: CR was observed in 65.2% of patients with DLBCL and in 67.9% of patients with FL, while CR/CRu was observed in 69.7% and 73.6% of patients, respectively. Rates of response in this study were in line with those observed in the Spanish “sister” study [17]. In line with results of randomised clinical trials that compared the SC and the IV routes of rituximab administration in patients with DLBCL [12] and FL [13], the median EFS, PFS, and OS were not estimable due to the low number of patients with events: at a median follow-up of 29.5 and 47.8 months in patients with DLBCL and FL, respectively, more than 70% of patients were event-free in the overall population and in both subgroups for EFS and PFS, and approximately 90% of patients in the overall population were alive. These data are in support of the adequacy of the pharmacokinetics of rituximab when administered by SC route.

The satisfactory safety profile and the efficacy of SC rituximab led to a high level of patients' satisfaction with treatment, as confirmed by high mean scores of the satisfaction with the injection, tolerability at the site of injection, expectations for injection, and interference of injections with daily activities at the end of both the induction and maintenance treatments.

However, caution should be exercised in the interpretation of efficacy data of this study due to the nonrandomised nature of the study and due to some limitations. First, the study included patients that received a previous full dose of rituximab IV as induction/maintenance without interruption, and thus patients who did not respond or progressed early in the course of treatment were excluded. Moreover, approximately 25% of enrolled patients were not evaluable for response and more than half of patients with DLBCL were at low-low/intermediate IPI risk. This might have led to the selection of a cohort of patients likely to have a good response to treatment.

More in general, the study was designed to mimic the common daily clinical practice, in which participants could be switched after at least one dose of IV rituximab plus other therapies. This means that the study did not allow standardization in terms of duration of previous rituximab administration or stratification by type and duration of chemotherapy. Patients were also followed according to local practice, which may vary across sites. Finally, although the study was not designed to compare the results in the two populations, there was no planned stratification between patients with DLBCL and FL, which has led to a markedly different distribution of the two groups across regions. This imbalance does not allow generalization of the results, as the safety and efficacy of rituximab may differ between patients with DLBCL and FL.

## 5. Conclusions

The data analysis of patients participating in this phase IIIb study in Italy showed that treatment with SC rituximab given to patients with DLBCL and FL previously treated with at least one dose of IV rituximab was associated with low risk of ARRs and satisfactory response in both groups of patients.

## Figures and Tables

**Table 1:** Disposition of patients.

	All patients (*N* = 159)	DLBCL (*N* = 73)	FL (*N* = 86)
Enrolled patients	159 (100.0%)	73 (100.0%)	86 (100.0%)
Analysed population	158 (99.4%)	72 (98.6%)	86 (100.0%)

Number of patients who completed final staging	67 (42.1%)	67 (91.8%)	NA
Prematurely discontinued on or before the final staging	8 (5.0%)	8 (11.0%)	NA

*Reason for discontinuation* ^*∗*^ *:*			
Progression of disease	2 (25.0%)	2 (25.0%)	
Adverse event	2 (25.0%)	2 (25.0%)	
Investigator decision	1 (12.5%)	1 (12.5%)	
Lost to follow-up	1 (12.5%)	1 (12.5%)	
Death	2 (25.0%)	2 (25.0%)	

Patients who discontinued the study	38 (24.1%)	13 (18.1%)	25 (29.1%)

*Reason for discontinuation* ^*∗*^ *:*			
Progression of disease	14 (36.8%)	7 (53.8%)	7 (28.0%)
Adverse event	12 (31.6%)	2 (15.4%)	10 (40.0%)
Consent withdrawn	2 (5.3%)	-	2 (8.0%)
Investigator decision	2 (5.3%)	1 (7.7%)	1 (4.0%)
Lost to follow-up	1 (2.6%)	1 (7.7%)	-
Death	2 (5.3%)	2 (15.4%)	-
Other reasons	5 (13.2%)	-	5 (20.0%)

Number of patients who continued to maintenance phase	77 (48.4%)	NA	77 (89.5%)

^*∗*^Percentages for the reasons for discontinuation are based on the number of patients who discontinued. Other percentages are calculated based on number of patients in the analysed population. *N*: number of patients; DLBCL: diffuse large B-cell lymphoma; FL: follicular lymphoma; NA: not applicable.

**Table 2:** Demographic and baseline characteristics of patients.

	All patients (*N* = 158)	DLBCL (*N* = 72)	FL (*N* = 86)
*Age (years)*			
Mean (SD)	58.7 (11.28)	59.7 (12.70)	57.8 (9.92)
Median (range)	59.5 (27–80)	61.0 (27–80)	56.5 (30–80)

*Age category, n (%)*			
≥18 and ≤64	107 (67.7%)	42 (58.3%)	65 (75.6%)
>64 and ≤80	51 (32.3%)	30 (41.7%)	21 (24.4%)

*Sex, n (%)*			
Male	86 (54.4%)	44 (61.1%)	42 (48.8%)
Female	72 (45.6%)	28 (38.9%)	44 (51.2%)

*Ethnicity, n (%)*			
Hispanic	9 (5.7%)	4 (5.6%)	5 (5.8%)
Non-Hispanic	131 (82.9%)	59 (81.9%)	72 (83.7%)
Not applicable	3 (1.9%)	1 (1.4%)	2 (2.3%)
Other	15 (9.5%)	8 (11.1%)	7 (8.1%)

*BMI (kg/m* ^*2*^)			
Mean (SD)	26.2 (4.81)	25.3 (4.40)	27.0 (5.02)
Median (range)	25.8 (16.5–47.6)	25.8 (16.6–36.2)	25.9 (16.5–47.6)

*ECOG performance status*			
Grade 0	107 (67.7%)	36 (50.0%)	71 (82.6%)
Grade 1	43 (27.2%)	30 (41.7%)	13 (15.1%)
Grade 2	7 (4.4%)	5 (6.9%)	2 (2.3%)
Missing	1 (0.6%)	1 (1.4%)	0 (0.0%)

*IPI score*			
Low risk	28 (17.7%)	28 (38.9%)	-
Low intermediate risk	15 (9.5%)	15 (20.8%)	-
High intermediate risk	17 (10.8%)	17 (23.6%)	-
High risk	12 (7.6%)	12 (16.7%)	-

*FLIPI Score*			
Low risk	25 (15.8%)	-	25 (29.1%)
Intermediate risk	32 (20.3%)	-	32 (37.2%)
High risk	29 (18.4%)	-	29 (33.7%)

*Grade of FL*			
1	9 (5.7%)	-	9 (10.5%)
2	45 (28.5%)	-	45 (52.3%)
3a	32 (20.3%)	-	32 (37.2%)

*N*: number of patients; DLBCL: diffuse large B-cell lymphoma; FL: follicular lymphoma; SD: standard deviation; BMI: body mass index; ECOG: Eastern Cooperative Oncology Group; IPI: International Prognostic Index; FLIPI: Follicular Lymphoma International Prognostic Index.

**Table 3:** Overall summary of adverse events. Data are number (%) of patients.

	All patients (*N* = 158)	DLBCL (*N* = 72)	FL (*N* = 86)
TEAEs	134 (84.8%)	61 (84.7%)	73 (84.9%)
Treatment-emergent SAEs	49 (31.0%)	26 (36.1%)	23 (26.7%)
ARRs	10 (6.3%)	3 (4.2%)	7 (8.1%)
Cutaneous and soft tissue ARRs (localised)	8 (5.1%)	1 (1.4%)	7 (8.1%)
Cutaneous and soft tissue ARRs (nonlocalised)	2 (1.3%)	2 (2.8%)	0 (0.0%)
Grade ≥3 TEAEs	74 (46.8%)	37 (51.4%)	37 (43.0%)
Grade ≥3 treatment-emergent SAEs	48 (30.4%)	25 (34.7%)	23 (26.7%)
Grade ≥3 ARRs	0 (0.0%)	0 (0.0%)	0 (0.0%)
Grade ≥3 infusion/injection-related reactions	0 (0.0%)	0 (0.0%)	0 (0.0%)
TEAEs leading to rituximab interruption or delay	33 (20.9%)	11 (15.3%)	22 (25.6%)
TEAEs leading to rituximab dose discontinuation	8 (5.1%)	2 (2.8%)	6 (7.0%)
TEAEs leading to chemotherapy dose modification	3 (1.9%)	2 (2.8%)	1 (1.2%)
TEAEs leading to chemotherapy dose discontinuation	4 (2.5%)	1 (1.4%)	3 (3.5%)
TEAEs leading to death	2 (1.3%)	2 (2.8%)	0 (0.0%)

*N*: number of patients; DLBCL: diffuse large B-cell lymphoma; FL: follicular lymphoma; TEAE: treatment-emergent adverse event; SAE: serious adverse event; ARR: administration-related reaction.

**Table 4:** Treatment-related TEAEs by preferred term. Data are number (%) of patients [number of events].

	All patients (*N* = 158)	DLBCL (*N* = 72)	FL (*N* = 86)
Neutropenia	14 (8.9%) [18]	5 (6.9%) [6]	9 (10.5%) [12]
Erythema	5 (3.2%) [5]	0 (0.0%) [0]	5 (5.8%) [5]
Leukopenia	5 (3.2%) [7]	1 (1.4%) [1]	4 (4.7%) [6]
Injection site erythema	3 (1.9%) [4]	0 (0.0%) [0]	3 (3.5%) [4]
Pyrexia	3 (1.9%) [3]	2 (2.8%) [2]	1 (1.2%) [1]
Lymphopenia	3 (1.9%) [5]	0 (0.0%) [0]	3 (3.5%) [5]
Influenza-like illness	2 (1.3%) [2]	0 (0.0%) [0]	2 (2.3%) [2]
Herpes virus infection	2 (1.3%) [2]	0 (0.0%) [0]	2 (2.3%) [2]
Neutrophil count decreased	2 (1.3%) [2]	2 (2.8%) [2]	0 (0.0%) [0]
Rash	2 (1.3%) [3]	1 (1.4%) [2]	1 (1.2%) [1]
Anaemia	1 (0.6%) [1]		1 (1.2%) [1]
Febrile neutropenia	1 (0.6%) [1]	1 (1.4%) [1]	
Hypoglubulinaemia	1 (0.6%) [1]	1 (1.4%) [1]	
Thrombocytopenia	1 (0.6%) [1]	1 (1.4%) [1]	
Diarrhoea	1 (0.6%) [1]	1 (1.4%) [1]	
Paraesthesia oral	1 (0.6%) [4]	1 (1.4%) [4]	
Administration site pain	1 (0.6%) [1]	1 (1.4%) [1]	
Inflammation	1 (0.6%) [1]		1 (1.2%) [1]
Injection site oedema	1 (0.6%) [1]		1 (1.2%) [1]
Injection site rash	1 (0.6%) [1]		1 (1.2%) [1]
Injection site swelling	1 (0.6%) [1]		1 (1.2%) [1]
Oedema	1 (0.6%) [1]		1 (1.2%) [1]
Hypertransaminasemia	1 (0.6%) [1]	1 (1.4%) [1]	
Folliculitis	1 (0.6%) [1]		1 (1.2%) [1]
Herpes zoster	1 (0.6%) [1]		1 (1.2%) [1]
*Klebsiella* infection	1 (0.6%) [1]	1 (1.4%) [1]	
*Pneumocystis jirovecii* infection	1 (0.6%) [1]		1 (1.2%) [1]
Pneumonia	1 (0.6%) [1]	1 (1.4%) [1]	
Upper respiratory tract infection	1 (0.6%) [1]		1 (1.2%) [1]
Vulvovaginitis	1 (0.6%) [1]		1 (1.2%) [1]
Gout	1 (0.6%) [1]		1 (1.2%) [1]
Hypokalaemia	1 (0.6%) [1]	1 (1.4%) [1]	
Hyponatraemia	1 (0.6%) [1]		1 (1.2%) [1]
Paraesthesia	1 (0.6%) [1]	1 (1.4%) [1]	
Chylothorax	1 (0.6%) [1]	1 (1.4%) [1]	

*N*: number of patients; TEAE: treatment-emergent adverse event; DLBCL: diffuse large B-cell lymphoma; FL: follicular lymphoma.

## Data Availability

The data used to support the findings of this study are included within the article.
